# Role of Cholecystokinin (*cck*) in Feeding Regulation of Largemouth Bass (*Micropterus salmoides*): Peptide Activation and Antagonist Inhibition

**DOI:** 10.3390/biology13080635

**Published:** 2024-08-20

**Authors:** Hualiang Liang, Haifeng Mi, Heng Yu, Dongyu Huang, Mingchun Ren, Lu Zhang, Tao Teng

**Affiliations:** 1 Key Laboratory of Integrated Rice-Fish Farming Ecology, Ministry of Agriculture and Rural Affairs, Freshwater Fisheries Research Center, Chinese Academy of Fishery Sciences, Wuxi 214081, China; 2 Tongwei Agricultural Development Co., Ltd., Key Laboratory of Nutrition and Healthy Culture of Aquatic Livestock and Poultry, Ministry of Agriculture and Rural Affairs, Healthy Aquaculture Key Laboratory of Sichuan Province, Chengdu 610093, China; 3 Wuxi Fisheries College, Nanjing Agricultural University, Wuxi 214081, China

**Keywords:** largemouth bass (*Micropterus salmoides*), cholecystokinin (*cck*), peptide activation, antagonist inhibition, regulation of feeding

## Abstract

**Simple Summary:**

Cholecystokinin (*cck*) is an important factor in regulating feed intake. According to a previous study, replacing fish meal (FM) in the diet of largemouth bass could downregulate the expression of the *cck* gene; however, the underlying regulatory mechanism remains unclear. Therefore, we investigated the effect of *cck* on feed intake and its potential mechanism of action via injecting exogenous CCK peptide and its receptor antagonist. This study’s results indicate that *cck* could regulate the feed intake of largemouth bass through regulating feeding-related genes in the brain and intestine. Furthermore, *cck* required binding with the receptor to inhibit feed intake more effectively in largemouth bass, and the binding effect of cholecystokinin receptor 1 (CCK1R) was better than that of cholecystokinin receptor 2 (CCK2R), which could lay a theoretical foundation for the study of fish feeding regulation.

**Abstract:**

This study investigated the role of cholecystokinin (*cck*) in the feeding regulation of largemouth bass (*Micropterus salmoides*) via peptide activation and antagonist inhibition. The results show that the *cck* gene was expressed in various tissues, with the highest expression level occurring in the brain. Feeding, continuous feeding, and refeeding after fasting could significantly improve the mRNA levels of *cck* in the brain. Moreover, the activation of *cck* via injecting an exogenous CCK peptide could inhibit feed intake by regulating the mRNA levels of anorexigenic and feed-promoting factors in the brain and intestine. Furthermore, the CCK peptide reduced feed intake; however, the presence of an antagonist (Ly225910-CCK1R and devazepide-CCK2R) could reverse this effect through regulating the mRNA levels of anorexigenic and feed-promoting factors in the brain and intestine. Treatment with devazepide + CCK (CCK2R) reversed feed intake more effectively than Ly225910 + CCK (CCK1R) treatment. In summary, *cck* could regulate the feed intake of largemouth bass through regulating feeding-related genes in the brain and intestine. In addition, *cck* required binding with the receptor to inhibit feed intake more effectively in largemouth bass, and the binding effect of CCK1R was better than that of CCK2R.

## 1. Introduction

The central nervous system in vertebrates receives and processes peripheral information regarding feed intake and nutritional status [1,2]. Feed is one of the most authoritative external signals that can induce feeding behavior and growth in fish [3,4]. Feed intake is influenced by neuroendocrine signaling (homeostasis regulation of the peripheral and central nervous systems) [5]. As in vertebrates, the brain is the main regulatory center for feed intake in fish and is regulated by central and peripheral feeding factors.

Cholecystokinin (*cck*) is an important factor in regulating feed intake. In mammals, CCK, a brain–gut peptide, is released in the brain and peripheral neurons in response to a meal to inhibit feed intake [6]. The first evidence of CCK’s involvement in feeding regulation in fish was found in a study on goldfish (*Carassius auratus*) [7]. Subsequently, CCK was reportedly associated with the regulation of feed intake in the spotted river puffer (*Tetraodon nigroviridis*) and Japanese flounder (*Paralichthys olivaceus*) [8], blunt snout bream (*Megalobrama amblycephala*) [9], yellowtail (*Seriola quinqueradiata*) [10], Schizothoracine fish (*Schizothorax prenanti*) [11], winter skate (*Raja ocellata*) [12], and white sea bream (*Diplodus sargus*) [13]. CCK has two classes of receptors, including the CCK1 (cholecystokinin receptor 1, CCK1R) and CCK2 (cholecystokinin receptor 2, CCK2R) receptors [14,15]. Notably, CCK-8 inhibits feed intake in mice via CCK1R [16]. Similarly, CCK affects feed intake mainly through CCK1R in Siberian sturgeon (*Acipenser baerii*) [17]; however, different CCK receptors may affect fish feeding due to differences in physiological factors. For instance, the feed composition and nutrient level can affect the expression of feeding genes in herbivorous and carnivorous fish [18,19,20]. Abasubong et al. [21] showed that dietary protein levels and fish meal (FM) had significant effects on the expression of cholecystokinin (*cck*) and neuropeptide Y (*npy*) genes in the brains of juvenile channel catfish (*Ictalurus punctatus*). The feed intake and the mRNA levels of *cck* and peptide YY (*pyy*) significantly decreased following the complete replacement of FM with soy protein concentrate in the intestine of dourado (*Salminus brasiliensis*) [22]. Similarly, replacing 45–90% of the FM with blood red opal powder significantly downregulated the mRNA levels of agoutine-associated protein (*agrp*), *npy*, and orexin genes in the hypothalamus of a hybrid grouper (*Epinephelus fuscoguttatus* × *Epinephelus lanceolatus*) [18].

Largemouth bass (*Micropterus salmoides*) is native to North America and was largely introduced to China in the 1980s. It is one of the most important freshwater fish produced in Chinese aquaculture due to its wide temperature tolerance, rapid growth rate, and versatility in adapting to different conditions [23,24]. As a carnivorous fish species, largemouth bass has a high demand for FM, constituting approximately 40–55% dry matter [25]. However, the price of FM continues to rise due to its unsustainable and increasing demand, thereby forcing the reduction of FM content in feed and the identification of other animal or even plant-based proteins as replacements, which may lead to the reduced feeding of farmed fish. According to a previous study, replacing FM in the diet of largemouth bass can downregulate the expression of the *cck* gene [26]; however, the underlying regulatory mechanism remains unclear. Therefore, we investigated the effect of *cck* on feed intake and its potential mechanism of action by injecting exogenous CCK peptide and its receptor antagonist. The present study lays a theoretical foundation for promoting fish feeding and growth via nutritional intervention through substituting FM. 

## 2. Materials and Methods

### 2.1. Experimental Fish

Healthy and uniform largemouth bass specimens were temporarily kept in floating cages (1 m × 1 m × 1 m) in outdoor ponds and indoor circulation farming systems (farming tanks with a height and diameter of 86 cm and 72 cm, respectively) for acclimatization. Commercial feed for largemouth bass from Wuxi Tongwei Aquatic Feed Co., Ltd. (Wuxi, China) (for which the protein and lipid levels were 48% and 12%, respectively) was given during this period to ensure normal fish growth.

### 2.2. Expression Levels of the cck Gene in Different Tissues

During the acclimatization, the largemouth bass specimens were fed at 8:00 a.m. daily to ensure their normal feeding and healthy state. After the temporary feeding experiment, four largemouth bass specimens (average weight: 69.40 ± 0.78 g) were anesthetized with 100 mg/L of MS-222, obtained from Abmole Bioscience Inc. (Houston, TX, USA). Tissues were then collected from the foregut, midgut, hindgut, liver, eye, spleen, heart, stomach, gill, kidney, and brain. The samples were put into a frozen tube and immediately transferred into a liquid nitrogen tank for quick freezing.

### 2.3. Changes in cck Gene Expression before and after Feeding

A total of 48 largemouth bass (average weight: 71.27 ± 0.07 g) were randomly divided into two feeding and nonfeeding groups. The fish in the feeding group were fed at 08:00 a.m. daily, while the nonfeeding group was not fed. The sampling time points of brain tissues were 0 h (with the feeding time at 08:00 a.m.), −1 h (1 h before feeding), +1 h, +3 h, +6 h, and +12 h (1, 3, 6, and 12 h after feeding, respectively). The samples were put into a frozen tube and immediately transferred into a liquid nitrogen tank for quick freezing.

### 2.4. Study of cck Gene Expression after Short-Term Fasting

A total of 45 largemouth bass (average weight: 69.73 ± 0.23 g) were randomly assigned to five experimental groups (feeding for 3 consecutive days, fasting for 3 consecutive days, feeding for 7 consecutive days, fasting for 7 consecutive days, and fasting for 7 consecutive days followed by feeding). Brain tissues were collected from these experimental groups and stored as mentioned above.

### 2.5. Treatment with CCK Peptide and CCK Peptide + Receptor Antagonists

The optimal doses of CCK peptide, the CCK1R antagonist (devazepide), and the CCK2R antagonist (Ly225910) were determined by observing the difference in the feed intake after injecting the respective molecules into the largemouth bass. CCK peptide was synthesized by GenScript Biotech Corporation (Nanjing, China) with a purity of ≥95.0% with the following amino acid sequence: MQTPEPTSVSCWQDSSPPGKVLCAETPQQTAKAAD. Ly225910 (GlpBio, Montclair, CA, USA) and devazepide (MedChemExpress, New York, NY, USA) were obtained from Shanghai Hongye Biotechnology Co., Ltd. (Shanghai, China) and Shanghai Haoyuan Biotechnology Co., Ltd. (Shanghai, China), respectively. Experiment I confirmed the appropriate dose of CCK peptide. The fish fasted for 24 h. Then, 144 fish were randomly divided into six groups (1 control and 5 experimental groups) with 24 fish each (average weight: 40.04 ± 0.02 g). The fish injected with 100 μL of normal saline were the control group. Those in the experimental groups were injected with 20, 50, 100, 150, and 200 ng/g of body weight (BW) of CCK peptide. Experiment II confirmed the appropriate dose of the CCK1R antagonist (devazepide) and the CCK2R antagonist (Ly225910). Based on previous studies, the initial doses of devazepide and Ly225910 were set as 0.1, 0.5, and 1.0 mg/kg BW [17,27,28,29]. The largemouth bass fasted for 24 h before the experiment. Then, 192 fish were randomly divided into eight groups (one control and seven experimental groups) with 24 fish each (average BW: 37.60 ± 0.05 g). The fish injected with 100 μL of normal saline were the control group. The fish in the seven experimental groups were injected with CCK peptide (with the optimal dose determined in Experiment I) and 0.1, 0.5, and 1.0 mg/kg BW of devazepide and Ly225910 each, respectively. The experimental fish were then put into a circulating culture tank for recovery. The fish were freely fed with excess feed for 1 h. The uneaten puffed pellet feed was collected and dried in an oven to measure the total feed intake [17,29]. According to the feed intake, the optimal dosages of CCK peptide, devazepide, and Ly225910 were determined for the follow-up experiment.

### 2.6. Expression of Feeding Genes in Brain and Intestine after CCK Peptide Injection

The largemouth bass fasted for 24 h before the experiment, and 80 fish (average weight: 36.09 ± 0.20 g) were randomly divided into two experimental groups of 40 fish each. The fish in the control and treatment groups were injected with 100 μL of normal saline and CCK peptide (at the optimal dose determined in Experiment I), respectively. After the treatment, eight fish were collected from each group at 0, 1, 3, and 6 h, anesthetized with MS-222 (100 mg/L), and immediately dissected. The brain and intestinal tissues were collected in frozen tubes and transferred into a liquid nitrogen tank for quick freezing. Subsequently, all the samples were stored at −80 °C to study the expression of *cck* and other feeding-related genes in the brain and intestine. Moreover, the optimal time taken for CCK peptide to show effect was determined.

### 2.7. Expression of Feeding Genes in Brain and Intestine after Treatment with CCK Peptide and Receptor Antagonists

Before the experiment, 32 largemouth bass fasted for 24 h (average weight: 37.12 ± 0.03 g) and were then randomly assigned to four groups of 8 fish each. The fish in the control group were injected with 100 μL of normal saline, and those in the first experimental group were injected with CCK peptide (at the optimal dose determined in Experiment I). The second and third experimental groups were injected with 50 μL of devazepide and Ly225910 (at the optimal doses determined in Experiment II), respectively, followed by injecting both groups with 50 μL of CCK peptide. After one hour, intestinal and brain tissues were collected from four fish anesthetized with MS-222. All the samples were stored at −80 °C for further use.

### 2.8. Real-Time Quantitative Fluorescence (qPCR) Analysis

The total RNA extraction from the liver and intestine samples employed the RNAiso Plus (Vazyme) reagent, obtained from Vazyme Biotech Co., Ltd. (Nanjing, China). The A260/280 value of 1.8–2.0 served as a standard for further qualitative and quantitative analyses using a Thermo Scientific NanoDrop 2000 spectrophotometer (Waltham, MA, USA). Based on the analysis of all the standard curves of the designed primers, the designed primer amplification efficiencies of genes were 98.1–99.5% and 0.989 < R^2^ < 0.998. The primers were sequenced using CFX96 Touch (Bio-Rad, Singapore), and the 2^−ΔΔCt^ method was used to calculate mRNA levels based on *β-actin* [30]. The primer details for gene amplification are shown in Table 1.

### 2.9. Statistical Analysis

The data were subjected to normality and homogeneity tests. One-way analysis of variance (ANOVA) was performed using SPSS (20.0) for data analysis, Tukey’s test was used for pairwise comparisons (*p* < 0.05), and the independent sample *t*-test was used for comparisons between two groups to analyze the results. Data values with different letter superscripts represent significant differences (*p* ˂ 0.05), while asterisks indicate significant differences between groups (*p* ˂ 0.05).

## 3. Results

### 3.1. Expression of cck Gene in Different Tissues

**Figure 1:**
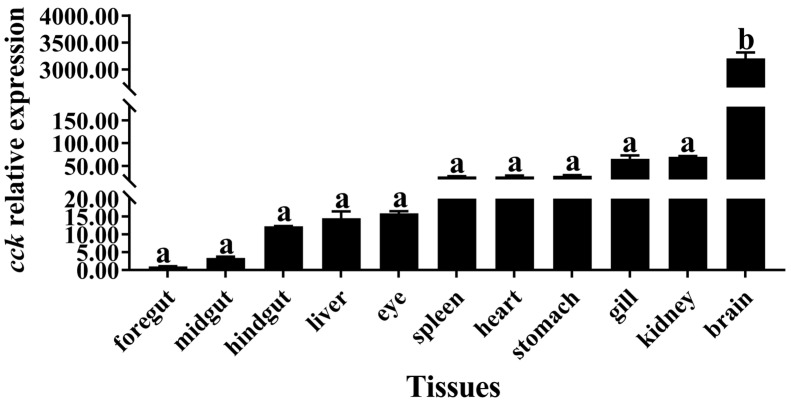
Relative expressions of *cck* in different tissues of largemouth bass (*Micropterus salmoides*). Data are presented as means ± standard error. The different superscripts of the means indicate significant differences.

The highest mRNA levels of *cck* were found in the brain; however, no significant differences were found in the foregut, midgut, hindgut, liver, eye, spleen, heart, stomach, gill, or kidneys (*p* > 0.05; Figure 1).

### 3.2. Relationship between cck Gene Expression and Feeding Regulation

**Figure 2:**
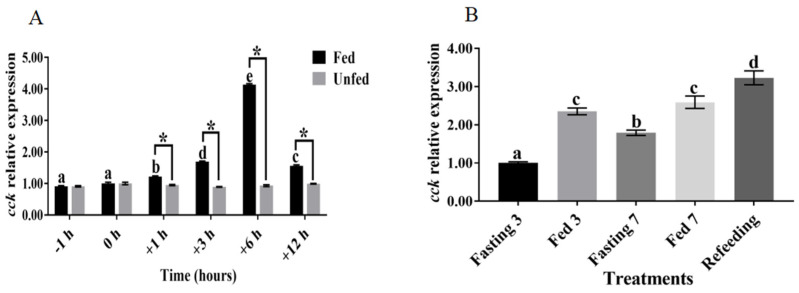
The relative expression of *cck* at different feeding time points of largemouth bass (*Micropterus salmoides*). Data are presented as means ± standard error. (**A**) Changes in brain *cck* gene expression before and after feeding. Different superscripts indicate a significant difference in relative *cck* expression over time. The asterisks indicate expression levels that were significantly different between fed and unfed treatments at each time point. (**B**) Study of *cck* gene expression in the brain after short-term fasting. Different superscripts indicate significant differences in relative *cck* expression between treatments.

The *cck* mRNA levels remained unchanged in the brain between the −1 h and 0 h feeding time points (*p* > 0.05); however, maximum levels were observed at the +6 h feeding time point (Figure 2A). Conversely, the levels were significantly decreased at the +12 h feeding time point (*p* < 0.05) (Figure 2A). Simultaneously, no significant changes in the *cck* expression levels were observed in the brains of the nonfeeding group at the different time points (*p* > 0.05) (Figure 2A). The results of the short-term fasting experiments revealed that the *cck* mRNA levels in the brain were significantly improved after 3 days of continuous feeding compared with those after continuous fasting (*p* < 0.05) (Figure 2B). Similarly, *cck* expression levels were significantly increased after 7 days of continuous feeding relative to continuous fasting for 7 days (*p* < 0.05) (Figure 2B). Moreover, the resumption of feeding after 7 days of fasting caused a significant increase in the mRNA levels of *cck* in the brain compared with the other treatment groups (*p* < 0.05) (Figure 2B).

### 3.3. Effects of Injecting Exogenous CCK Peptide on Feeding Regulation

**Figure 3:**
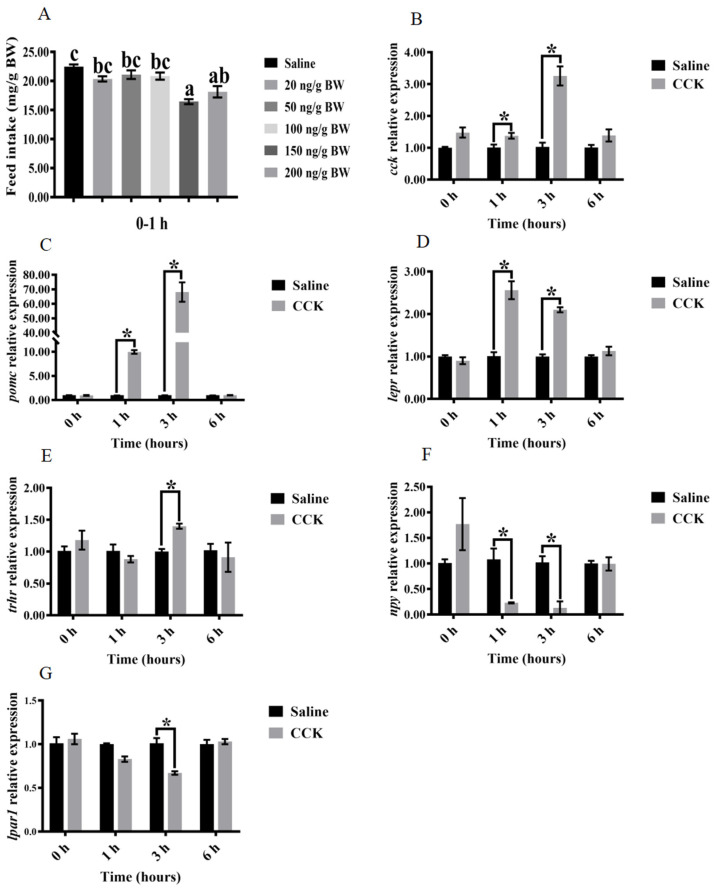
Effects of the injection of exogenous CCK peptide on feed intake and the relative expression of feeding-related genes in the brain of largemouth bass (*Micropterus salmoides*). (**A**) Feed intake. (**B**) *cck*. (**C**) *pomc*. (**D**) *lepr*. (**E**) *trhr*. (**F**) *npy*. (**G**) *lpar1*. Feed intake (mg/g body weight) = 1000 × total weight of feeding weight/total weight of fish. Data are presented as means ± standard error. (**A**) Study of feed intake after fish were injected with different concentrations of CCK peptide. Different superscripts indicate significant differences in feed intake between treatments. (**B**–**G**) Changes in brain feeding-related gene expression after the injection of exogenous CCK peptide and saline. The asterisks indicate expression levels that were significantly different between treatments at each time point.

**Figure 4:**
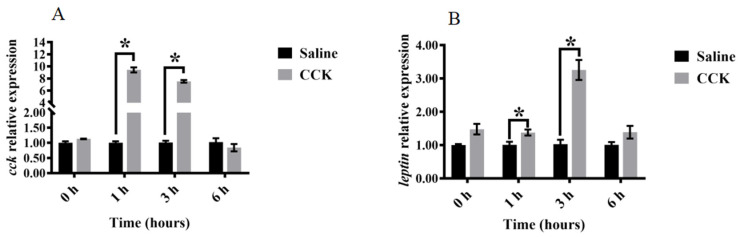
Effects of the injection of exogenous CCK peptide on feeding-related genes in the intestine of largemouth bass (*Micropterus salmoides*). (**A**) *cck*. (**B**) *leptin*. Data are presented as means ± standard error. The asterisks indicate expression levels that were significantly different between treatments at each time point.

Compared with the control group, the feed intake was significantly decreased in the groups treated with 150 and 200 ng/g BW of CCK peptide after feeding for 0–1 h (*p* < 0.05) (Figure 3A). Specifically, the lowest feed intake was observed at 150 ng/g BW (*p* < 0.05) (Figure 3A). In contrast, the mRNA levels of *cck* and anorexia genes, including *pomc* and *lepr*, in the brain were significantly increased at 1 and 3 h after injecting CCK peptide (*p* < 0.05) (Figure 3B–D). Similarly, the mRNA levels of *trhr* were significantly increased at 3 h after CCK peptide administration (*p* < 0.05) (Figure 3E). Conversely, the mRNA levels of feed-promoting genes in the brain, including *npy* and *lpar1*, were significantly decreased after 3 h of injecting CCK peptide (*p* < 0.05) (Figure 3F,G). Furthermore, significantly higher mRNA levels of *cck* and *leptin* were found in the intestine after 1 and 3 h of injecting CCK peptide compared with those in the control group (*p* < 0.05) (Figure 4A,B).

### 3.4. Effects of Co-Injecting Exogenous CCK Peptide and CCK Receptor Antagonists on Feeding Regulation

**Figure 5:**
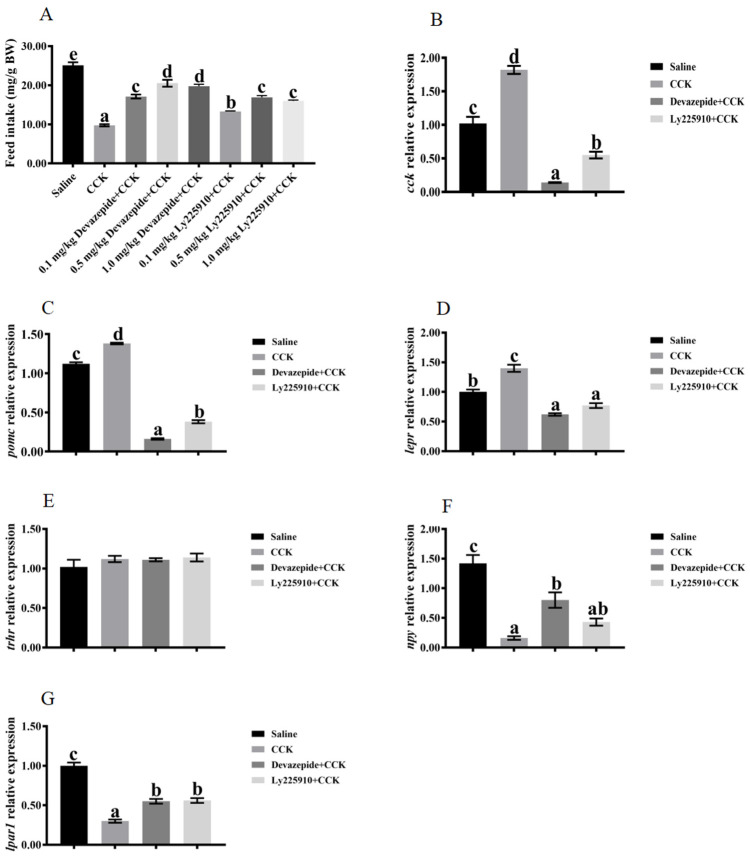
Effects of the co-injection of exogenous CCK peptide and CCK receptor antagonists on feed intake and the relative expression of feeding-related genes in the brain of largemouth bass (*Micropterus salmoides*). (**A**) Feed intake. (**B**) *cck*. (**C**) *pomc*. (**D**) *lepr*. (**E**) *trhr*. (**F**) *npy*. (**G**) *lpar1*. Data are presented as means ± standard error. The different superscripts of means indicate significant differences.

**Figure 6:**
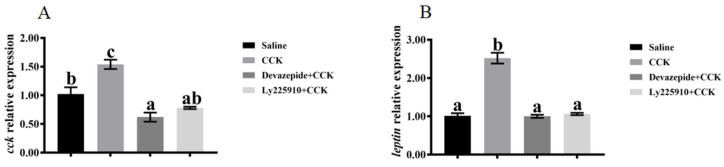
Effects of the co-injection of exogenous CCK peptide and CCK receptor antagonists on feeding-related genes in the intestine of largemouth bass (*Micropterus salmoides*). (**A**) *cck*. (**B**) *leptin*. Data are presented as means ± standard error. The different superscripts of means indicate significant differences.

The feed intake was significantly reduced in the group administered with CCK peptide compared with the control group (*p* < 0.05) (Figure 5A). Subsequently, co-injection of CCK peptide and CCK receptor antagonists reversed the CCK peptide-induced feed intake reduction (*p* < 0.05) (Figure 5A). The feed intake in the groups treated with 0.5 and 1.0 mg/kg of devazepide + CCK peptide was significantly higher than in those treated with 0.1 mg/kg of devazepide + CCK peptide (*p* < 0.05) (Figure 5A). Similarly, the feed intake in the groups treated with 0.5 and 1.0 mg/kg of Ly225910 + CCK peptide was significantly higher than in those treated with 0.1 mg/kg of Ly225910 + CCK peptide (*p* < 0.05) (Figure 5A). Additionally, the reversal of feed intake was significantly higher in the group treated with 0.5 mg/kg of devazepide + CCK peptide relative to that with 0.5 mg/kg of Ly225910 + CCK peptide (*p* < 0.05) (Figure 5A). Significantly higher mRNA levels of *lepr*, *cck*, and *pomc*, and lower mRNA levels of *npy* and *lpar1*, were found in the brains of fish injected with CCK peptide compared with the control group (*p* < 0.05) (Figure 5B–G). Moreover, significantly downregulated mRNA levels of *cck*, *pomc*, and *lepr* in the brain were observed in the groups co-injected with devazepide + CCK and Ly225910 + CCK (*p* < 0.05) compared with those injected with CCK peptide alone (Figure 5B–D). Meanwhile, the devazepide + CCK group significantly improved the mRNA levels of *npy* in the brain (*p* < 0.05) (Figure 5F), and the devazepide + CCK group and the Ly225910 + CCK group significantly improved the mRNA levels of *lpar1* in the brain (*p* < 0.05) (Figure 5G). However, no significant change was observed in the mRNA levels of *trhr* (Figure 5E). The fish injected with CCK peptide showed significantly improved mRNA levels of intestinal *cck* and *leptin* compared with the control group (*p* < 0.05) (Figure 6A,B). In contrast, significantly lower mRNA levels of *cck* and *leptin* were found in the intestine in the co-injection of devazepide + CCK group and the Ly225910 + CCK group compared with those treated with CCK peptide (*p* < 0.05) (Figure 6A,B).

## 4. Discussion

The mechanisms and roles of CCK receptors in the feeding regulation of largemouth bass have been rarely studied. In our previous study, the mRNA level of *cck* was significantly downregulated after replacing FM in the diet of largemouth bass [26]. This study further investigated the role and mechanism of *cck* in feeding regulation. The results reveal that *cck* was expressed in multiple tissues of largemouth bass, with the highest mRNA levels in the brain. *cck* is distributed mainly in the brain of various carnivorous fish, including Siberian sturgeon [29] and Yangtze sturgeon (*Acipenser dabryanus*) [33], omnivorous fish, including goldfish [34], and herbivorous fish, including grass carp (*Ctenopharyngodon idellus*) [35]. These findings suggest that the brain is an important regulatory center for feed intake [36,37], and that *cck* might be a crucial regulatory factor for feed intake in the brain of largemouth bass.

Pre- and post-feeding and fasting tests are often used to investigate the effect of a factor on feed intake. The results of a previous study suggested that *cck* levels were high up to 5 h after a meal in humans [38]. In addition, a study on Siberian sturgeon reported that significantly higher mRNA levels of *cck* were found in the brain after feeding than in the nonfed group after 1 and 3 h [29]. Similarly, the *cck* mRNA levels in the brain were significantly increased after feeding in channel catfish (*Ictalurus punctatus*) [39] and Atlantic salmon (*Salmo salar*) [40]. Collectively, these results suggest that feed intake could increase the mRNA levels of *cck* in the brain and that *cck* might act as a satiating factor. Our results of the fasting experiment showed significantly higher mRNA levels of *cck* in the brain after continuous feeding for 3 to 7 days relative to fasting. In addition, a study on cyprinid fish (*Schizothorax prenanti*) demonstrated significantly upregulated expression of *cck* in the hypothalamus of fish after continuous feeding for 7 days compared with fasting [11], and similar results were reported in Siberian sturgeon [29]. The underlying mechanism could be the possible attenuation of *cck* gene expression in the brain after fasting, thereby inhibiting the satiety signal and leading to a hunger response in largemouth bass. Combined with the results of the pre- and post-feeding and fasting experiments, it was preliminarily concluded that the hungry or full state of largemouth bass might modulate *cck* expression in the brain to regulate feed intake.

Regulating feed intake has been previously investigated by injecting exogenous peptides in grass carp [41], goldfish [42], and channel catfish [43]. In this study, different doses of CCK peptide (20, 50, 100, 150, and 200 ng/g BW) were injected into largemouth bass. Compared with the normal group, fish injected with 150 and 200 ng/g BW of CCK peptide showed a significantly reduced feed intake within 1 h. Consequently, 150 ng/g BW was selected as the optimal dose of CCK peptide for further experiments. Previous studies have reported different optimal doses of CCK peptide for different fish. For instance, 100 ng/g BW of CCK8 was administered to Siberian sturgeon [17]. CCK is a kind of brain–intestinal peptide [44]. In this study, significantly higher mRNA levels of *cck* and *leptin* in the intestine were also observed after 1 and 3 h of injecting CCK peptide, compared with those injected with normal saline. Furthermore, Volkoff et al. [45] found a synergistic relationship between *leptin* and *cck*. In mammals, leptin acts on the hypothalamus to exert an anorexia response [46], which inhibits *npy* and stimulates *pomc* expression [47,48]. In fish, leptin released from tissues reaches the hypothalamus and binds to *lepr*. The binding of *leptin* and *lepr* results in the downregulation of *npy* and the upregulation of *pomc* expression, leading to reduced feed intake [49]. In this study, besides the variations in the mRNA levels of intestinal *cck* and *leptin*, the mRNA levels of *cck*, *pomc*, and *lepr* in the brain were significantly increased at 1 and 3 h after injecting CCK peptide. Meanwhile, the dietary stimulating factor *npy* was significantly downregulated. These results indicate that exogenous CCK peptide could cause anorexia through upregulating the mRNA levels of anorexia-related genes in the brain and intestine while downregulating feed-promoting genes in the brain. Consequently, the probable action time of CCK peptide is 1–3 h after injection. However, whether CCK acts as a target site to directly affect other feeding-related genes or coregulates feed intake with other genes needs further investigation.

We injected CCK peptide and its receptor antagonist to determine the optimal dose of the receptor antagonists (devazepide and Ly225910), estimated to be 0.5 mg/kg, to further analyze CCK’s mechanism. In addition, the mRNA levels of feeding-related genes in the brain and intestine of fish after 1 h of treatment were determined. Fish injected with CCK peptide showed significantly improved mRNA levels of *cck*, *pomc*, and *lepr*, and decreased levels of *npy* and *lpar1* in the brain compared with the control group. In addition, increased mRNA levels of intestinal *cck* and *leptin* were observed. Moreover, the group injected with CCK peptide + the antagonists reversed the expression levels of feeding-related genes. Specifically, the feed intake and mRNA levels of related genes were significantly higher in the group injected with devazepide + CCK relative to that with Ly225910 + CCK, suggesting that CCK needed to bind to the receptor to inhibit the feed intake in largemouth bass, and the effect of binding to CCK1R was better than that of CCK2R. CCK1R antagonists inhibited feed intake in mice, whereas CCK2R antagonists had no effect [16]. Additionally, treatment with a CCK1R activator reduced feed intake in Göttingen minipigs [50]. Therefore, CC1R might be a potential target for the future regulation of feed intake in largemouth bass.

## 5. Conclusions

In summary, *cck* regulated the feed intake in largemouth bass through regulating feeding-related genes in the brain and intestine. This study provides a theoretical basis and technical means for alleviating the problem of decreased feed intake after fish meal substitution via regulation of *cck*.

## Figures and Tables

**Table 1:** qPCR primer sequences.

Genes	Forward Primer (5′-3′)	Reverse Primer (5′-3′)	Accession No./Reference
*cck*	TAAAGGGAAGTCACGGCTCATAC	CGGTTATTCTCAACAGACCCTGA	XM_038724067.1
*cck1r*	CCGTGCTGGTGAGGAACAGG	GCCAGTGCCGAAGACGAAGT	[31]
*cck2r*	GCGCGCCCATCTCCTTCATC	GCCTCCCTCTTCCTGCACCA	[31]
*leptin*	CTTTTCATTCACGTGTTTCGCTG	CCTCTGACTGCAAACAACCTTAC	MN887534.1
*lepr*	TTGTCCCACAAAGAAGACACAGA	AGTGTAAAATCAGCTCAGCCTCA	XM_038715328.1
*pomc*	GTGAAAGGAGAGGGAAGAGACAG	AGAACACGACATCAACTCTGGAA	XM_038725660.1
*trhr*	GCCACAGAGTAAGCAGAAT	TCACATCACATCACATCACA	XM_038705006.1
*npy*	GTCATCAGTGTTGGCTCCACCTCA	CAACATGCCCTCCTCCACTTTACT	[32]
*lpar1*	CCACCATAACGAACACTCT	GCTCATCATCAACTCTACCT	XM_038733709.1
*β-actin*	ATGCAGAAGGAGATCACAGCCT	AGTATTTACGCTCAGGTGGGG	AF253319.1

Note: *cck*, cholecystokinin; *cck1r*, cholecystokinin receptor 1; *cck2r*, cholecystokinin receptor 2; *lepr*, leptin receptor; *pomc*, proopiomelanocortin; *trhr*, thyrotropin-releasing hormone receptor; *npy*, neuropeptide Y; *lpar1*, lysophosphatidic acid receptor 1; *β-actin*, beta-actin.

## Data Availability

Data is contained within this article.
